# The Impact of Abdominoplasty vs Non-abdominoplasty on Weight Loss in Bariatric and Non-bariatric Malaysian Patients: A Multicentre Retrospective Study

**DOI:** 10.7759/cureus.23996

**Published:** 2022-04-09

**Authors:** Wan Ratmaazila Wan Makhtar, Nurul Syazana Mohamad Shah, Siti Muyassarah Rusli, Arman Zaharil Mat Saad, Wan Azman Wan Sulaiman

**Affiliations:** 1 Reconstructive Sciences Unit, Universiti Sains Malaysia School of Medical Sciences, Kubang Kerian, MYS; 2 Plastic and Reconstructive Surgery Unit, Hospital Canselor Tuanku Muhriz Universiti Kebangsaan Malaysia (UKM), Cheras, MYS; 3 Plastic and Reconstructive Surgery Unit, Management and Science University Medical Centre, Shah Alam, MYS

**Keywords:** weight loss, non-bariatric, bariatric, non-abdominoplasty, abdominoplasty

## Abstract

Background and aims

Weight loss associated with abdominoplasty remains controversial as to whether it is contributed by the procedure alone, or if there are other patient factors. Therefore, we evaluated the relationship between weight loss in pre- and post-abdominoplasty and compared the weight loss of those who do not undergo abdominoplasty in bariatric and non-bariatric patients.

Methods

This study measured weight changes at designated time points in four different groups comprising 98 patients. The groups were abdominoplasty after bariatric surgery (group A), patients with abdominoplasty alone (B), bariatric surgery alone (group C), and diet alone (group D). Results are compared within and between the groups.

Results

Patients in groups A and B (patients who had abdominoplasty regardless with or without bariatric surgery) had a significant weight loss after six months (mean difference=10.70kg, 95% CI=4.05, 17.34, p<0.001) compared to pre abdominoplasty weight. Patients in group B were statistically significant (mean difference=4.01kg, 95% CI=0.92, 7.10, p=0.007) with 3.60kg weight reduction (4.59%). Patients in group A had clinically significant weight reduction (4.45kg, 5.14%) but it was not statistically significant (p>0.05) and had no significant additional weight reduction as compared to non-abdominoplasty (group C) patients (p=0.650). Patients in group C had a statistically significant difference from those in group D (F [1.00, 48.00] = 8.56, p=0.005) with an average weight loss of 3.60kg (4.59%) vs 2.56kg (2.8%) respectively.

Conclusions

All patients with abdominoplasty had significant weight loss after six months. Bariatric patients did not experience significant additional weight loss with abdominoplasty. Weight reduction after abdominoplasty alone was greater than diet alone.

## Introduction

A nutritional status does not only reflect health status, but also the social and economic circumstances of the population group. Body mass index (BMI), waist, and calf circumference are the parameters that are being used to assess a nutritional status as an essential element of the health of the population. The National Health and Morbidity Survey (NHMS) 2019 has concluded that the national prevalence of overweight, obesity and abdominal obesity were 30.4%, 19.7%, and 52.6% respectively [1]. The current prevalence of overweight, obesity, and abdominal obesity showed an increase of 30.0%, 17.7%, and 48.6% respectively as compared to the previous study in 2015 [2]. The prevalence of overweight in adults aged 18 years and above in Malaysia is still lower than the global prevalence of 39%, but higher in the obesity group by an increase of 4.7% from the global prevalence [3]. The high prevalence of overweight and obesity in Malaysia is contributed to by many factors. These include ‘westernization’ of global eating habits brought by fast food outlets in the last decade, the increasingly sedentary lifestyle existence among populations, and inherited metabolic predisposition to a fatness ‘thrifty genotype’ [4-6].

There are many therapeutic strategies that could be delivered to promote weight loss. These include promoting a healthy lifestyle among populations such as walking and exercising, intervention programs conducted at the workplace, and targeting specific groups such as school children and middle-aged women. However, these programs are usually temporary and unsustainable [7-9]. The responsibility to control this epidemic not only involves the health sector but also other sectors or non-governmental organizations (NGOs) to conduct a healthier living and provide a health-promoting environment [10].

Nonpharmacological or conventional obesity treatment at primary health care centers was not associated with short or long-term weight loss, while anti-obesity drugs may result in an 8-12% weight reduction over two years [10]. For the past three decades, only bariatric surgical procedures such as open or laparoscopic gastric bypass surgery have been useful to produce permanent weight loss [11]. In Malaysia, bariatric surgery has been increasing in popularity preceded by special dietary advice for potential bariatric patients.

Abdominoplasty is a body contouring surgery that involves removing large abdomen pannus that first began in the 1800s. Later, several techniques of abdominoplasty were developed to achieve more inconspicuous scar and optimum body contour results. It was generally accepted as body contouring surgery instead of weight loss surgery. In Malaysia, abdominoplasty was chosen both due to post-bariatric surgery, post-diet or non-bariatric, or diet patients seeking better body contour. A previous retrospective pilot study pointed out that abdominoplasty performed on patients with BMI >24.5kg/m2 was linked to sustaining weight loss at one year and the contributing factors were increased satiety and amount of fat resected [12]. Moreover, patients who underwent body contouring procedures after bariatric surgery had significantly better long-term weight loss than a matched cohort of patients [13]. Previous studies have also reported that the patients had a better quality of life [14,15]. Other previous studies found blood changes such as leptin, low-density lipoprotein (LDL), and triglyceride are also associated with abdominoplasty [16-18].

With the astonishing numbers of the prevalence of overweight and obesity as reported in the literature, measures to curb this epidemic in the country were limited to lifestyle modification and bariatric surgery. This study investigated the relationships between weight loss in bariatric patients with abdominoplasty and bariatric patients alone, and with patients who underwent abdominoplasty alone within six months. We also evaluate the association of the weight loss in patients who underwent abdominoplasty alone with patients who were on a special weight management diet. We finally evaluated the association of satiety in all patients who underwent abdominoplasty as compared to all patients who did not undergo abdominoplasty within six months.

## Materials and methods

Subjects

This is a retrospective cohort study with case controls using data from three main hospitals in Malaysia: Hospital Kuala Lumpur, Hospital Universiti Kebangsaan Malaysia, and Hospital Universiti Sains Malaysia. The total numbers of patients recruited in this study were 98 based on sample size estimation on comparison of k group means using one-way ANOVA with two-sided equality [19]. The duration of this study was two years. Data was collected through a random sampling method. Participants were selected based on the fulfilments of inclusion criteria obtained from the surgical registry. No blinding was done as this was a retrospective cohort. Bias was controlled in this study as there was no pre-determined specific weight loss target for each group, but rather considered significant if proven statistically. This study was approved by the Human Research Ethics Committee (HREC) Universiti Sains Malaysia, Kubang Kerian, Kelantan, Malaysia (USM/JEPeM/17090415) and the Medical Research and Ethics Committee, Ministry of Health of Malaysia (NMRR-18-26-39762).

Inclusion and exclusion criteria

Inclusion criteria were patients who underwent bariatric surgery followed by abdominoplasty within two years later (group A), patients who underwent abdominoplasty alone only (group B), patients who underwent bariatric surgery alone only (group C), and patients who were put on weight loss diet plan for obesity for at least six months without having surgery (group D). Any patients with documented underlying malignancy and chronic lower gastrointestinal inflammatory diseases such as Crohn’s disease or ulcerative colitis, physical disability to execute standard exercise protocol, pregnancy within the duration of the study, or any documented established psychiatric patients were excluded.

Subject recruitment

Three departments or units were involved in this study, namely the Bariatric Unit, Dietitian Unit, and the Plastic & Reconstructive Department. From the Plastic & Reconstructive Department, recruitment was done through screening of operative records and unit records tracing of all patients who underwent abdominoplasty procedure, which includes both patients who had a history of bariatric surgery and those who do not. From the Bariatric Unit, recruitment of patients started by screening the census of the Bariatric Unit’s patient records. These were patients who underwent bariatric surgery one year ago and did not proceed with an abdominoplasty. The bariatrics patients included in this study were those who underwent either open or laparoscopic gastric bypass or sleeve gastrectomy. From the Dietitian Unit, patients who were on a special weight management diet plan were recruited. After patient recruitment, selected patients were divided according to two main groups: ‘abdominoplasty’ and ‘non-abdominoplasty’ patients. The two main groups were further subdivided into bariatric and non-bariatric, resulting in four end groups:

Group A 

Abdominoplasty and bariatric: These were patients who underwent bariatric surgery and underwent abdominoplasty within two years after bariatric surgery.

Group B

Abdominoplasty and non-bariatric: These were patients who never had any bariatric surgery but underwent an abdominoplasty. They were also not on any meal replacement therapy before or after the procedure.

Group C

Non-abdominoplasty bariatric: These were patients from the obesity clinic who had more than one year post-bariatric surgery but had no abdominoplasty done.

Group D 

Non-abdominoplasty non-bariatric: These were patients from the dietitian clinic who did not undergo any bariatric surgical intervention or abdominoplasty procedure. They were on meal replacement therapy or a special weight management diet plan for at least six months.

Methods

The baseline weight measurement was taken on Day 0 during the first clinic visit to the Plastic Surgery clinic for bariatric treatment for groups A, B, and D. While for group C, the baseline was taken upon follow-up one year post-bariatric surgery. However, their pre-bariatric surgery weight was also taken a day before, not to compare to other groups but to assess the weight declination trend within 18 months within the group. For groups A and B, a second weight measurement was taken during ward admission, one day before their surgery. For groups C and D, the second weight measurement was taken three months after the first weight measurement. For groups A and B, the fourth weight measurement was taken three months after the abdominoplasty procedure. The last weight measurement was taken six months after the abdominoplasty procedure for groups A and B, and six months after the first weight measurement for groups C and D. During this time, the patient was asked simple questions regarding their satiety, physical exercises, health food intake and any newly diagnosed illness that may contribute to weight changes.

Data was entered and analyzed using the predictive analysis software IBM SPSS Version 27 (IBM Corp., Armonk, NY, USA). To compare within and between groups, two-way mixed ANOVA was used. A chi-square test was used to evaluate satiety in patients who had abdominoplasty and a Pearson correlation was used to evaluate the correlation between the weight of pannus and weight loss post abdominoplasty.

## Results

Data from a total of 98 patients were collected. This included 23 patients for group A, 25 patients for group B, 25 patients for group C and 25 patients for group D. Table 1 shows the grouping of the patients according to the type of treatment received. From the results, we found that bariatric patients mostly had abdominoplasty within the late two years after bariatric surgery instead of around one year post-bariatric surgery, with an average at 19 months. For groups A, B, and D, the time point taken for weight measurements can be fairly compared between groups. However, the first two time points for group C should be disregarded when comparing with other groups, although it may be worth it to compared to its own group to show its weight trend.

**Table 1:** Timepoint according to groups ^a^Bariatric surgery with abdominoplasty ^b^Non-bariatric surgery with abdominoplasty ^c^Bariatric surgery with non-abdominoplasty ^d^Non-bariatric surgery with non-abdominoplasty i.e., diet alone T= Time point

Group	T1	T2	T3	T4	T5
A^a^	1st clinic appointment prior to abdominoplasty procedure	Day of admission before abdominoplasty	Immediate post- abdominoplasty	3 months appointment	6 months appointment
B^b^	1st clinic appointment	Day of admission before abdominoplasty	Immediate post- abdominoplasty	3 months appointment	6 months appointment
C^c^	Pre-bariatric surgery	Post-bariatric surgery at 6 Months	Post-bariatric surgery at 12 Months	Post-bariatric surgery at 15 months	Post-bariatric surgery at 18 Months
D^d^	N/A	N/A	The first appointment for diet consultation	3 months appointment	6 months appointment

Association of weight loss in bariatric patients who underwent abdominoplasty with the weight loss of non-bariatric patients who underwent abdominoplasty within six months (group A vs group B)

Using a two-way mixed ANOVA, (F) with a Greenhouse-Geisser correction determined that the mean weight showed significant difference within the time frame of first clinic appointment, pre-abdominoplasty, post abdominoplasty, three and six months (F [4.00,44.00]=12.22, p<0.001). Post hoc tests using Bonferroni correction revealed that overall patients who underwent abdominoplasty regardless of with or without bariatric surgery exhibited the most weight loss three months post abdominoplasty as compared to pre abdominoplasty (mean difference=10.70kg, 95% CI=4.05,17.34) which is statistically significant (p <0.001). For within-group weight reduction, total weight reduction in group A was 13.72kg (14.3% reduction from baseline), while in group B was 7.91kg (9.6% reduction from baseline). The mean difference was significant (p<0.001). However, there was no statistically significant weight difference between these two groups (Mean difference=8.998kg, F [1.00,46.00]=1.67, p=0.203). Similarly, there was no significant interaction between weight reduction and the group who underwent abdominoplasty regardless of with or without bariatric surgery (p=0.558). Therefore, for between-group weight reduction, the difference was not significant at all time points of weight measurements (p>0.05). Both groups exhibited an almost similar reduction trend in body weight at six months follow-up after abdominoplasty procedure.

We also compared the weight difference from the last three time points (T4 - T5) separately to exclude the influence of pre-operative weight measurement from the analysis. A two-way mixed ANOVA with a Greenhouse-Geisser correction, (F) determined that the mean weight differed significantly within the time frame of post abdominoplasty, three months and six months for overall patients who underwent abdominoplasty regardless of with or without bariatric surgery (F [1.00,47.00]=5.51, p=0.007).

The post hoc test for group A revealed that there was no significant mean difference of weight between all three measurements points (p>0.05). For group B, post hoc tests using the Bonferroni correction revealed that weight loss at six months (mean difference=4.01kg, 95% CI=0.92,7.10, p=0.007) were higher than at three months (mean difference=2.39kg, 95% CI=0.69,4.10, p=0.003) if compared with post abdominoplasty weight. For weight reduction after abdominoplasty, the total weight at the final point of mean weight measurement group A after abdominoplasty was 82.09kg at six months which showed a 4.45kg reduction from 86.54kg (5.14% weight reduction since immediate abdominoplasty), while in group B was 74.80kg from 78.40kg with 3.60kg reduction (4.59% weight reduction since immediate abdominoplasty) from the immediate weight after abdominoplasty (from T3). The mean difference was statistically not significant with p>0.050.

There was no statistically significant weight difference between the group (patients who underwent abdominoplasty after bariatric surgery and patients who underwent abdominoplasty alone) within six months (mean difference=7.67kg, F [1.00,46.00]=1.48, p=0.231). Similarly, there was no significant interaction between weight and group (p=0.913).

Association of weight loss of bariatric patients who underwent abdominoplasty with bariatric surgery and the weight loss of bariatric patients who did not undergo abdominoplasty within six months (group A vs group C)

Using a two-way mixed ANOVA, (F) with a Greenhouse-Geisser correction determined that the mean weight showed significant difference within the time frame of first clinic appointment, pre abdominoplasty, post abdominoplasty, three and six months (F [4.00,43.00]=32.02, p<0.001). Post hoc tests using Bonferroni correction revealed that bariatric patients who underwent abdominoplasty and bariatric patients who did not undergo abdominoplasty exhibited the most weight loss six months post abdominoplasty as compared to pre abdominoplasty and 18 months as compared to pre bariatric surgery, respectively (mean difference=22.01kg, 95% CI=13.58,30.43) which is statistically significant (p<0.001). For weight reduction after abdominoplasty in bariatric patients (group A), the total weight at the final point of weight measurement at six months was 82.09kg, which showed a 10.94kg reduction from 95.81kg (13.2% reduction since first clinic TCA), while in group C was 84.87kg from 115.16kg with 30.29kg reduction from pre bariatric surgery (26.3% reduction since T1). Therefore, pre-weight for A and C was 115.15(SD=27.43) and 95.81(SD=37.49) and eventually decreased at the end of study time points to 82.09(SD=23.01) and 84.87(SD=19.02) respectively. The mean difference was not significant (p>0.050) except for comparison at T1 (p=0.046).

There was no statistically significant weight difference between patients who underwent abdominoplasty after bariatric surgery and patients who underwent bariatric surgery alone within six months and 12 to 18 months respectively (mean difference=-9.22kg, F [1.00,46.00]=1.54, p=0.221). There was a significant interaction between weight and group (p<0.001) and the difference was significant at the first clinic appointment only (p=0.046) but at other times of measurements, the mean weights were almost similar.

For group C with no abdominoplasty done, weight first dramatically reduced within the initial 12 months post bariatric surgery, but further reduced slowly after 12 months follow-up. Total mean weight reduction among those who underwent bariatric surgery after 18 months follow-up was 30.28kg (26.3% reduction from before bariatric surgery).

After 12 months of bariatric surgery, patients who underwent bariatric surgery alone (group C) experienced further weight reduction of 6.29kg (5.46% additional weight reduction after 12 months [T3 until T5]). For patients who underwent abdominoplasty after bariatric surgery (group A), there was also a further decline in weight loss after abdominoplasty whereby total weight reduction in group A was 13.72kg (14.3% reduction from before abdominoplasty).

To compare between groups, we separately analyzed the result of the last three time points (T3 until T5) as we disregard pre-bariatric weight measurements from group C and we disregard pre abdominoplasty weight from group A. A two-way mixed ANOVA with a Greenhouse-Geisser correction, (F) determined that the mean weight of all patients in group A and C have differed significantly within the time frame (F [2.00,45.00]=3.24, p=0.048). Post hoc tests using Bonferroni correction revealed for group C and group A, there was no significant difference in mean weight between the time of measurements (all p>0.050). There was also no statistically significant weight difference between patients who underwent abdominoplasty and bariatric surgery within six months and patients who underwent bariatric surgery only after 12 months (F [1.00,46.00]=0.21, p=0.650). Plus, there was also no significant interaction effect between body weight and groups (F [2.00,45.00]=0.59, p=0.589).

For weight reduction after six months since immediate abdominoplasty for group A, and at 18 months post-bariatric surgery since 12 months bariatric surgery for group C (T5 from T3), the total weight at the final point of weight measurement for group A after abdominoplasty was 82.09kg at six months, which showed 4.45kg reduction from 86.54 (5.14% reduction since immediate abdominoplasty), while in group C was 84.87kg from 91.06kg with 6.19kg reduction (7.3% reduction since 12 months after bariatric surgery). The mean difference was not significant (p>0.050). For group A with abdominoplasty done, weight loss increased dramatically after six months follow-up. For group C with bariatric surgery only, the reduction was less drastic eventually.

Association of weight loss of patients who underwent abdominoplasty alone and weight loss of patients on a special weight management diet (group B vs group D)

A two-way mixed ANOVA with a Greenhouse-Geisser correction, (F) determined that the mean weight differed significantly within the time frame since the patient's first appointment to the respective unit clinic (F [2,23.0]=16.52, p<0.001). Hence, for within-group weight reduction, total weight reduction in group B was 7.91kg (9.5% reduction from the first appointment), while in group D was 2.6kg (2.8% reduction from the first appointment).

We also compared separately the weight difference of the last three time points of both groups B and D. A two-way mixed ANOVA with a Greenhouse-Geisser correction, (F) determined that the mean weight differed significantly within the time frame (F [2.00,48.00]=20.75, p<0.001). Then, there was a statistically significant weight difference between group B (patients who underwent abdominoplasty alone) and group D (patients on a special weight management diet) within six months post abdominoplasty and special diet started respectively (F [1.00,48.00]=8.56, p=0.005). While the weight of patients on a special diet (group D) was 90.38kg from 92.94kg, a weight reduction of 2.56kg (2.8% weight reduction six months after special diet), the weight of patients in group B was 74.80kg from 78.40kg with 3.60kg reduction (4.59% weight reduction since immediate abdominoplasty) from the immediate weight after abdominoplasty. There was no significant interaction effect between body weight and groups (F [2.00,48.00]=0.58, p=0.560). There was a similar trend of reducing weight in both groups in non-bariatric patients who underwent abdominoplasty with those who did not during the follow-up duration.

Correlation of post-operative weight loss with amount of fat removed in all patients who underwent abdominoplasty within six months

The correlation between the amount of fat removed and post-operative weight loss is shown in Table 2. A Pearson correlation, (r) was run to determine the relationship between postoperative weight loss with amount of fat (pannus) removed in all patients (group A and group B) who underwent abdominoplasty within six months. There was a weak, positive correlation between pannus weight and post-operative weight loss, which was statistically significant as in the graph below (r=0.32, n=48, p=0.03).

**Table 2:** Correlation between amount of fat removed and post-operative weight loss *Significant at p<0.05 n= number of pannus resected and measured r= Pearson correlation

Independent variable	Dependent variable	n	r	r^2^	p value
Pannus weight	Post-operative weight loss	48	0.32	0.10	0.03*

Association of satiety in all patients who underwent abdominoplasty as compared to all patients who did not undergo abdominoplasty within six months

**Figure 1:**
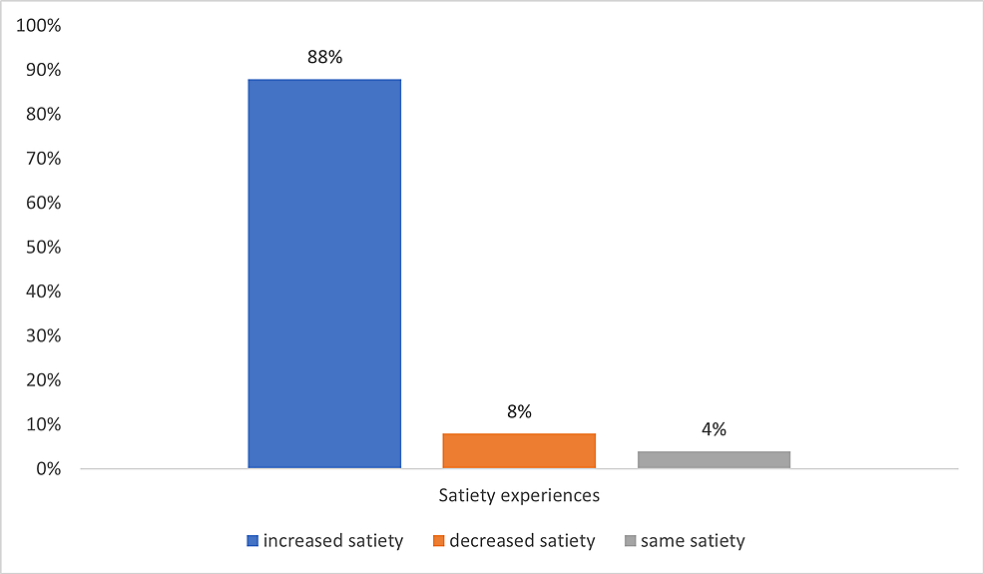
Experience of satiety for bariatric patients (group C)

For bariatric patients who underwent abdominoplasty (group A), 47.8% of patients (n=11) experienced the same satiety, 39.1% of patients (n=9) had an increase in satiety and 13% of patients (n=3) had a decrease in satiety as compared to prior to the operation. For bariatric patients who underwent bariatric surgery alone (group C), 88.0% of patients (n=22) experienced increased satiety, 8.0% of patients (n=2) had a decrease in satiety, and 4% of patients (n=1) had the same satiety as compared prior to operation as shown in Figure 1.

There was a significant association between satiety and abdominoplasty in bariatric patients (χ2=13.9, df=2, p=0.001) as shown in Table 3. For comparison of satiety in those without bariatric surgery, there was no significant association between abdominoplasty and satiety (p=0.82) as shown in Table 4.

**Table 3:** The association between satiety and undergoing abdominoplasty in bariatric patients *Significant at p<0.05 aBariatric surgery with abdominoplasty ^b^Bariatric surgery with non-abdominoplasty n= number of participants χ2= Chi square test df= Degree of freedom

Group	Satiety, n (%)			χ^2^	df	p value
	Same	Decrease	Increase			
^a^A	11 (47.8)	3 (13.0)	9 (39.1)	13.9	2	0.001*
^b^C	1 (4.0)	2 (8.0)	22 (88.0)			

**Table 4:** The association between satiety and undergoing abdominoplasty in non-bariatric patients *Significant at p<0.05 ^a^Non-bariatric surgery with abdominoplasty ^b^Non-bariatric surgery with non-abdominoplasty

Group	Satiety, n (%)			χ^2^	df	P value
	Same	Decrease	Increase			
^a^B	16 (64.0)	2 (8.0)	7 (28.0)	0.4	2	0.82
^b^D	14 (56.0)	3 (12.0)	8 (32.0)			

As for non-bariatric patients who underwent abdominoplasty alone (group B), 16% of patients (n=16) experienced the same satiety, 28% of patients (n=7) had an increase in satiety, and 8% of patients (n=2) had the same satiety as compared to prior to operation. For patients who underwent diet alone (group D), 56% of patients (n=14) experienced the same satiety, 32% of patients (n=8) had an increase in satiety, and 12% of patients (n=3) had the same satiety as compared prior to operation.

## Discussion

Abdominoplasty has long been accepted conventionally as body contouring surgery. The benefits of the procedure go beyond aesthetics, such as improved postural changes [20] and improved quality of life [15,21]. People who undergo body contouring surgery after bariatric surgery are also more satisfied with their appearance than those who do not undergo the procedure. The results of this study support observation of weight loss in patients as another benefit for those who had abdominoplasty procedures [12].

The number of bariatric surgeries in Asia has increased since 2005 [22], increasing the demand for body contouring surgery due to excess skin after massive weight loss. However, despite the many benefits of abdominoplasty procedures, not many medical insurance companies provide coverage for procedures related to body enhancing, as it is perceived as an aesthetic need rather than a medical requirement. One may wonder if weight loss after an abdominoplasty procedure in bariatric patients is contributed by the bariatric surgery effect rather than the body contouring surgery itself. We found that all patients who underwent abdominoplasty experienced a statistically significant weight loss (mean difference=10.70kg, 95% CI=4.05,17.34, p<0.001). The mean weight reduction that can be achieved can be up to 9% to 14% reduction from baseline weight (p<0.001).

Our results show that when comparing those who underwent abdominoplasty alone (group B) and those who underwent abdominoplasty after bariatric surgery (group A), both groups exhibit an almost similar significant increment trend in body weight loss from the follow-up (Table 2). However, the weight trend of six months after immediate abdominoplasty shows only a statistically significant weight reduction (p=0.03) in patients with abdominoplasty alone but not clinically significant in patients who had abdominoplasty after bariatric surgery (mean difference=4.01kg, 95% CI=0.92,7.10, p=0.007) with a mean weight reduction of 3.6kg (4.59% reduction from weight immediately after abdominoplasty). Therefore, we conclude that bariatric surgery does not significantly improve weight loss (mean difference=7.67kg, F [1.00,46.00]=1.48, p=0.231) as significantly as abdominoplasty alone. 

Bariatric patients are often advised for body contouring surgery after massive weight reduction post-bariatric surgery. The removal of extra subcutaneous tissue will definitely trim off some of the excess weight for more amount of weight loss after abdominoplasty [13]. However, we do not observe any patients with body contouring surgery when comparing results of weight loss in both bariatric groups (group A and group C). All bariatric patients regardless of abdominoplasty or not experienced significant weight reduction within our study time (F [2.00,45.00]=3.24, p=0.048). However, there is no significant effect on weight loss when compared with those who do not (F [1.00,46.00]=0.21, p=0.650).

There is a limitation for this result, as the weight of the bariatric group with no abdominoplasty is all measured after 12 months exactly, while those who underwent abdominoplasty within two years (mostly get the procedure done more than 15 months post-bariatric surgery). Most post-bariatric surgery patients will be recommended for a body contouring surgery as it is found that weight stabilization is usually after the first year [23].

Many of our patients are delayed for more than two years due to personal preferences and late appointment and surgery dates given as the procedure is not considered a priority as compared to other cases such as malignancy that need earlier surgical dates.

Coincidentally, we observed that the weight for those who underwent bariatric surgery mostly started to stabilize after 15 months post-bariatric surgery, that is 0.6kg reduction at 18 months compared to 15 months vs 5.69kg at 15 months compared to at 12 months (T5 minus T4 vs T4 minus T3).

Nevertheless, with abdominoplasty patients also get other benefits such as better body image and better postural changes. With the removal of excess skin after massive weight loss, it is easier for these patients to do physical activity and exercises, further contributing to more weight loss if not maintaining them in the long run. Furthermore, even though it is statistically insignificant, the mean weight reduction for those who underwent abdominoplasty after bariatric surgery in our study is 5.14% since immediate abdominoplasty, which is clinically significant and considered an improvement in weight-related morbidity from baseline for an individual intervention [24]. Clinical significance defines the extent of the result of weight reduction that was meaningful in relation to the treatment. 

From this study, we know that bariatric surgery does not play an effect on patients who experience weight loss after abdominoplasty (mean difference=7.67kg, F [1.00,46.00]=1.48, p=0.231). We found that there is a marked difference between the group who underwent abdominoplasty alone (group B) with those who are on a special diet for the weight management program (group D) (F [2.00,48.00]=20.75, p<0.001). These two groups also show the statistical significance of net weight (F [1.00,48.00]=8.56, p=0.005). Therefore, we observe that weight reduction is more prominent in those who underwent abdominoplasty than in those who do not. We found that patients who are on a special diet (group D) had a weight reduction to 90.38kg from 92.94kg, which is a mean weight reduction of 2.56kg (2.8% weight reduction six months after special diet). Meanwhile, patients in group B had their weight down to 74.80kg from 78.40kg, which totals up to 3.60 kg mean weight reduction (4.59% weight reduction) since immediate abdominoplasty. Therefore, we can conclude that abdominoplasty shows more weight reduction in patients who did abdominoplasty alone until six months follow-up as compared to patients with special diet plans (mean difference=1.19kg, F [1.00,48.00]=8.77, p=0.005).

Though abdominoplasty should not be considered as a quick fix to get a slimmer and better body contour, the results suggest that the procedure may have an impact on patients’ discipline and routine post-operatively as they are probably more motivated and more disciplined in keeping their body in shape considering the investments and ordeal they had to go through than those who are only on a special weight management diet who had nothing to lose whether or not they follow the regime. Those who seek abdominoplasty most probably came from a higher economic status background, which could also contribute to the weight loss, as higher socioeconomic status is found to be an independent predictor for weight loss in patients who seek plastic surgery post-bariatric surgery [25].

Our results also found that in patients who underwent abdominoplasty, there is a weak correlation between the weight of pannus removed and weight loss after the procedure (r=0.32, n=48, p=0.03). However, pannus weight accounted for only 10% of the total variation in postoperative weight loss, which indicates that there are other factors that contributed to the weight loss (r2=0.10). Suggested other factors that may contribute to weight loss include diet, exercise, and motivations.

Hormonal changes after removal of subcutaneous tissue have been found to have an impact on weight [26]. Leptin, a hormone secreted from adipocytes, regulates body weight and acts on the brain for food intake regulation. When secreted, it sends signals to the hypothalamus to reduce appetite and increase energy expenditure. However, in obese patients, this hormone is overexpressed leading to leptin resistance hence, the food intake becomes unregulated and uncontrollable. In abdominoplasty, the removal of adipose tissue removes the amount of leptin, and the decrease of excessive leptin concentration contributes to decreasing appetite and weight reduction. This effect however is only temporary and will return back from its temporary state [26]. This probably contributes to our result where the first weight measurement at three months is more drastic as compared to the second weight measurement post abdominoplasty.

With regards to satiety, our result found only bariatric groups with 39.1% (n=9) of patients from this group who underwent abdominoplasty experienced increased satiety (χ2=13.9, df=2, p=0.001). While in the non-bariatric group, 28% of patients (n=7) experienced increased satiety after abdominoplasty, which does not have statistical significance (p=0.82). Therefore, we could not comment on the association of satiety with abdominoplasty as reported by Fuller et al. [12] as our study is considered short term. We would like to suggest that other factors also contribute to weight loss instead of solely satiety such as motivations, diet, and exercise.

## Conclusions

All patients who underwent bariatric surgery (with or without abdominoplasty) had significant weight loss. All patients who underwent abdominoplasty will experience a statistically significant weight loss (9%-14%) from baseline. We found a clinically significant (5.4%) mean weight reduction after abdominoplasty in bariatric surgery patients. Abdominoplasty does contribute to weight loss more significantly than diet alone but does not play a role in satiety. Thus, we conclude that abdominoplasty is not only a body contouring procedure but is an important tool for weight reduction in the well-motivated patient. However, abdominoplasty should not be recommended as a surrogate for bariatric surgery but as an adjunct to it.
